# Mirikizumab Improves Quality of Life in Patients With Moderately-to-Severely Active Ulcerative Colitis: Results From the Phase 3 LUCENT-1 Induction and LUCENT-2 Maintenance Studies

**DOI:** 10.1093/crocol/otad070

**Published:** 2023-11-07

**Authors:** Bruce E Sands, Brian G Feagan, Theresa Hunter Gibble, Kristina A Traxler, Nathan Morris, William J Eastman, Stefan Schreiber, Vipul Jairath, Millie D Long, Alessandro Armuzzi

**Affiliations:** Dr Henry D. Janowitz Division of Gastroenterology, Icahn School of Medicine, Mount Sinai, NY, USA; Alimentiv, Inc., London, Ontario, Canada; Western University, London, Ontario, Canada; Eli Lilly and Company, Indianapolis, IN, USA; Eli Lilly and Company, Indianapolis, IN, USA; Eli Lilly and Company, Indianapolis, IN, USA; Eli Lilly and Company, Indianapolis, IN, USA; Department Internal Medicine I, University Hospital Schleswig-Holstein, Kiel University, Kiel, Germany; Western University, London, Ontario, Canada; Division of Gastroenterology and Hepatology, University of North Carolina, Chapel Hill, NC, USA; IBD Center, IRCCS Humanitas Research Hospital, Rozzano, Milan, Italy; Department of Biomedical Sciences, Humanitas University, Pieve Emanuele, Milan, Italy

**Keywords:** Mirikizumab, ulcerative colitis, quality of life

## Abstract

**Background:**

Mirikizumab, an anti-IL-23p19 antibody, demonstrated efficacy in phase 3, randomized, double-blind, placebo-controlled LUCENT-1 (induction/NCT03518086) and LUCENT-2 (maintenance/NCT03524092) ulcerative colitis (UC) studies. We evaluated the effect of mirikizumab on quality-of-life (QoL) outcomes in these studies.

**Methods:**

In LUCENT-1, 1162 patients with moderately-to-severely active UC were randomized 3:1 to receive mirikizumab 300 mg intravenous or placebo every 4 weeks (Q4W) for 12 weeks. In LUCENT-2, mirikizumab induction responders (*N* = 544) were re-randomized 2:1 to receive mirikizumab 200 mg subcutaneous or placebo Q4W through week (W) 40 (W52 of treatment). QoL was assessed at W12 and W52 using patient-reported outcomes. Treatments were statistically compared using analysis of covariance model (continuous outcomes) and Cochran–Mantel–Haenszel test (binary outcomes).

**Results:**

At W12 and W52, mirikizumab showed significant improvement in Inflammatory Bowel Disease Questionnaire (IBDQ) total and domain scores (*P* < .001); 36-Item Short Form Health Survey (SF-36) Physical Component Summary (PCS), Mental Component Summary (MCS), and domain scores (*P* < .05); EQ-5D-5L scores (*P* < .001); Work Productivity and Activity Impairment Questionnaire (UC) scores (*P* < .05); Patient Global Rating of Severity (*P* < .001); and Patient Global Rating of Change (*P* < .01) scores. A significantly higher proportion of mirikizumab-treated patients achieved IBDQ response (W12: 72.7% vs 55.8%; W52: 79.2% vs 49.2%; *P* < .001), IBDQ remission (W12: 57.5% vs 39.8%; W52: 72.3% vs 43.0%; *P* < .001), and clinically important improvements in PCS (W12: 50.6% vs 41.5%; W52: 61.9% vs 36.9%; *P* < .01) and MCS (W12: 44.2% vs 37.8%; W52: 51.2% vs 34.6%; *P* < .05) scores.

**Conclusions:**

Mirikizumab improved QoL in patients with moderately-to-severely active UC in phase 3 LUCENT-1 and LUCENT-2 studies.

**Clinical trials registration number:**

LUCENT-1: NCT03518086; LUCENT-2: NCT03524092

## Graphical Abstract

**Graphical Abstract:**
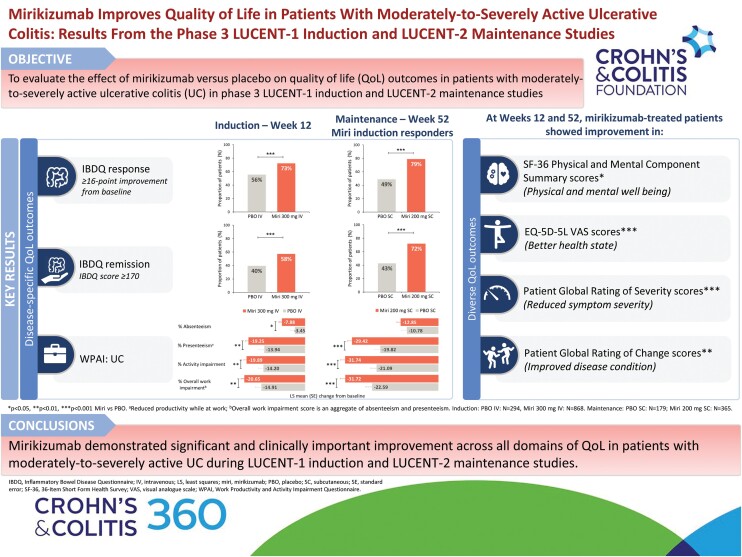

## Introduction

Ulcerative colitis (UC) is a chronic inflammatory bowel disease (IBD) characterized by mucosal inflammation of the colon. The disease exhibits a relapsing–remitting course; symptoms include diarrhea, rectal bleeding (RB), bowel urgency, and tenesmus.^1,2^

Patients with UC experience increased morbidity across all quality-of-life (QoL) domains. Moderately-to-severely active UC has been shown in previous studies to be associated with anxiety and depression,^3,4^ impaired social interactions, leisure activities, and work productivity.^5,6^ Thus, in addition to inducing and maintaining clinical remission, improvement in QoL is acknowledged as an important treatment goal for patients with UC.^7–9^

The limitations of conventional and existing therapies used for the management of UC include intolerance, lack or loss of response, or increased risks of infections or cancer.^9–11^ Mirikizumab is a humanized immunoglobulin G4 (IgG4) monoclonal antibody that inhibits interleukin (IL)-23 by binding to an epitope on the p19 subunit.^12^ In a phase 2 study (NCT02589665) in patients with UC, mirikizumab improved QoL relative to placebo as assessed by Inflammatory Bowel Disease Questionnaire (IBDQ) scores and Medical Outcomes Study 36-Item Short Form Health Survey (SF-36) Physical and Mental Component Summary (PCS and MCS, respectively) scores and domain scores at Weeks 12 and 52. Response rates for clinically meaningful improvements in IBDQ, PCS and MCS scores, and IBDQ remission were also higher in mirikizumab-treated patients at Weeks 12 and 52.^13^ In the phase 3, LUCENT-1 induction (NCT03518086) and LUCENT-2 maintenance (NCT03524092) studies in moderately-to-severely active UC, mirikizumab demonstrated efficacy across clinical, symptomatic, endoscopic, and histologic measures of disease, even after the failure of conventional immunosuppressives, biologic therapies, and/or tofacitinib.^14^

Herein, we present the effect of mirikizumab versus placebo on QoL outcomes in phase 3 LUCENT-1 and LUCENT-2 studies.

## Methods

### Study Design and Population

LUCENT-1 and LUCENT-2 are phase 3, multicenter, randomized, double-blind, parallel-arm, placebo-controlled studies. The studies included adult patients (aged ≥18 and ≤80 years) with moderately-to-severely active UC at screening (modified Mayo score [MMS] of 4–9 with an endoscopic subscore ≥2) who had failed (inadequate response/loss of response/intolerance) conventional therapy (corticosteroid, immunomodulators [6-mercaptopurine, azathioprine]), biologic therapy (anti-TNF antibody, vedolizumab), or Janus kinase (JAK) inhibitor (tofacitinib).

Detailed study design, eligibility criteria, and endpoints for LUCENT-1 and LUCENT-2 studies have been published previously.^14^

Study protocols and informed consent forms were approved by the ethical review board supervising each site. The study was compliant with International Conference on Harmonization Good Clinical Practice guidelines, Declaration of Helsinki, and Council for International Organizations of Medical Sciences International Ethical Guidelines. Written informed consent was provided by all patients.

### Randomization and Treatments

In the LUCENT-1 induction study, patients were randomized 3:1 to receive mirikizumab 300 mg or placebo intravenously (IV) every 4 weeks (Q4W) up to Week 12. Mirikizumab induction responders (patients who achieved ≥2 points and ≥30% decrease from baseline in MMS and had ≥1 point decrease from baseline in the RB subscore or an RB score of 0/1) at Week 12 (LUCENT-1) were re-randomized 2:1 to receive mirikizumab 200 mg or placebo subcutaneously Q4W up to Week 40 in the LUCENT-2 maintenance study. LUCENT-1 and LUCENT-2 together comprised a total of 52 weeks of continuous mirikizumab treatment, with Week 12 representing the end of the induction study and the start (Week 0) of the 40-week maintenance study.

Detailed randomization stratification criteria have been reported previously.^14^

### Study Outcomes and Assessments

Effect of mirikizumab on QoL was assessed using validated patient-reported outcome (PRO) measures. At Weeks 12 and 52, prespecified secondary endpoints included assessment of change from baseline in (1) IBDQ total and domain scores^15,16^; (2) SF-36 (Version 2) PCS, MCS, and domain scores^17,18^; (3) EQ-5D-5L visual analog scale (VAS)^19,20^; and (4) Work Productivity and Activity Impairment Questionnaire Ulcerative Colitis (WPAI:UC) score.^21,22^ IBDQ response (≥16-point improvement from baseline^15^) and remission (IBDQ score ≥170^23^) rates and SF-36 PCS and MCS minimal clinically important difference (MCID) response (≥5-point improvement from baseline^24^) rates were also evaluated at Weeks 12 and 52.

Prespecified exploratory endpoints included assessment of (1) Patient Global Rating of Severity (PGRS) up to Weeks 12 and 52 and (2) Patient Global Rating of Change (PGRC) at Weeks 12 and 52.

A detailed description of PROs is provided in Supplementary Table 1. For all the secondary and exploratory endpoints, responses were collected electronically at respective time points using an eDiary (PGRS) or a tablet device.

### Statistical Analyses

The statistical analyses were performed using SAS® Version 9.4 or higher.

Modified Intent-to-Treat (mITT) population included all randomized patients who received any amount of study treatment excluding patients impacted by the electronic clinical outcome assessment transcription error in Poland and Turkey.^14^

Patient demographics and baseline characteristics were summarized by treatment for the mITT population. Descriptive statistics were used to summarize continuous variables and frequency counts and percentages were used to summarize categorical variables.

All PRO analyses for LUCENT-1 were carried out in the mITT population. For LUCENT-2, all PRO analyses were performed in the subpopulation of patients who responded to mirikizumab induction therapy at Week 12. Baseline for both LUCENT-1 and LUCENT-2 studies was defined as the last nonmissing assessment recorded on or prior to the date of the first study drug administration at Week 0 in LUCENT-1.

For prespecified continuous endpoints, treatments were compared (mirikizumab versus placebo) using analysis of covariance (ANCOVA) model (for PGRC, ANCOVA as observed was used). Type III sums of squares for least squares (LS) means were reported for each treatment group. The ANCOVA model included treatment, baseline value, and stratification factors in the model. The LS mean difference, standard error, *P*-value, and 95% confidence interval (CI), unless otherwise specified, were reported. Missing data were imputed using modified baseline observation carried forward (mBOCF) method: Patients had their last post-baseline value carried forward, with the exception that patients who discontinued due to an adverse event had their baseline value carried forward.

Cochran–Mantel–Haenszel (CMH) test was used to compare the proportion of patients who achieved binary endpoints (PCS and MCS MCID response rates, and IBDQ response and remission rates) in the two treatment groups, while adjusting for the stratification factors. Estimated common risk differences with 95% CI (calculated using Mantel–Haenszel–Sato method^25^) and *P*-value (calculated using CMH) were reported. Missing data were imputed using nonresponder imputation (NRI) method.

The reported PRO measures were not included in the multiplicity-controlled testing scheme. Reported *P*-values are unadjusted for multiple testing and should not be interpreted as confirmatory.

## Results

### Patient Disposition and Characteristics

In the LUCENT-1 study, 1162 patients comprising the mITT population were randomized to mirikizumab 300 mg IV (*N* = 868) or placebo IV (*N* = 294).^14^ In the LUCENT-2 study, 544 mirikizumab clinical responders were re-randomized to mirikizumab 200 mg SC (*N* = 365) or placebo SC (*N* = 179).^14^ Patient demographics and baseline disease characteristics were generally balanced between the two treatment groups in the LUCENT-1 and LUCENT-2 studies (Table 1).

**Table 1.** Baseline demographics and disease characteristics—mITT.

		Induction		Maintenance Miri induction responders	
		PBO IV (*N* = 294)	Miri 300 mg IV (*N* = 868)	PBO SC (*N* = 179)	Miri 200 mg SC (*N* = 365)
Age (years), mean (SD)		41.3 (13.81)	42.9 (13.94)	41.2 (12.80)	43.4 (14.22)
Male, n (%)		165 (56.1)	530 (61.1)	104 (58.1)	214 (58.6)
BMI (kg/m^2^), mean (SD)		24.5 (5.05)	25.0 (5.39)	24.8 (5.18)	24.8 (5.39)
Duration of UC (years), mean (SD)		6.9 (6.95)	7.2 (6.75)	6.7 (5.61)	6.9 (7.10)
Baseline disease location, *n* (%)	Left-sided colitis	188 (64.2)	544 (62.7)	119 (66.5)	234 (64.1)
Modified Mayo Score category, *n* (%)	Moderate (4–6)	138 (47.1)	404 (46.5)	77 (43.0)	181 (49.6)
	Severe (7–9)	155 (52.9)	463 (53.3)	102 (57.0)	184 (50.4)
Total Mayo Score category, *n* (%)	Moderate (6–9)	186 (66.0)	519 (62.9)	108 (63.2)	224 (64.4)
	Severe (10–12)	93 (33.0)	297 (36.0)	61 (35.7)	119 (34.2)
Prior UC therapy, *n* (%)	Prior biologic or tofacitinib failure	118 (40.1)	361 (41.6)	64 (35.8)	128 (35.1)
Baseline UC therapy, *n* (%)	Corticosteroid	113 (38.4)	351 (40.4)	68 (38.0)	135 (37.0)
	Immunomodulator	69 (23.5)	211 (24.3)	39 (21.8)	78 (21.4)
IBDQ total score, mean (SD)		127.9 (35.26)	131.4 (33.04)	129.4 (31.94)	133.9 (33.16)
IBDQ subscores, mean (SD)	Bowel symptoms	37.6 (10.79)	38.9 (9.95)	37.8 (9.19)	39.5 (10.15)
	Systemic symptoms	18.1 (5.80)	18.7 (5.65)	18.3 (5.54)	19.0 (5.73)
	Emotional function	52.1 (14.81)	52.9 (13.99)	52.8 (14.02)	53.8 (13.81)
	Social function	20.2 (8.08)	20.9 (7.58)	20.5 (7.36)	21.6 (7.60)
SF-36, mean (SD)	MCS	43.47 (10.070)	43.95 (10.230)	43.31 (10.139)	44.68 (9.881)
	PCS	41.23 (8.284)	42.36 (7.885)	42.68 (8.050)	42.44 (7.758)
WPAI:UC employment status, *n* (%)	Yes	173 (59.7)	532 (62.1)	120 (67.4)	224 (62.0)
	No	117 (40.3)	325 (37.9)	58 (32.6)	137 (38.0)
Overall work impairment score^a^, mean (SD)		50.0 (28.13)	47.8 (25.81)	50.4 (25.55)	46.5 (26.46)
EQ-5D-5L VAS, mean (SD)		53.4 (20.59)	56.2 (19.16)	56.7 (18.48)	56.3 (18.77)
PGRS, mean (SD)		4.3 (0.82)	4.2 (0.83)	4.3 (0.77)	4.2 (0.85)

BMI, body mass index; IBDQ, Inflammatory Bowel Disease Questionnaire; IV, intravenous; MCS, Mental Component Summary; miri, mirikizumab; mITT, modified intent-to-treat; PBO, placebo; PCS, Physical Component Summary; PGRS, Patient’s Global Rating of Severity; SC, subcutaneous; SD, standard deviation; SF-36, 36-Item Short Form Health Survey; VAS, visual analog scale; UC, ulcerative colitis; WPAI:UC, Work Productivity and Activity Impairment Questionnaire:Ulcerative Colitis.

^a^Overall work impairment score is an aggregate of absenteeism and presenteeism.

### Patient-Reported Outcomes

#### Inflammatory Bowel Disease Questionnaire

**Figure 1.**
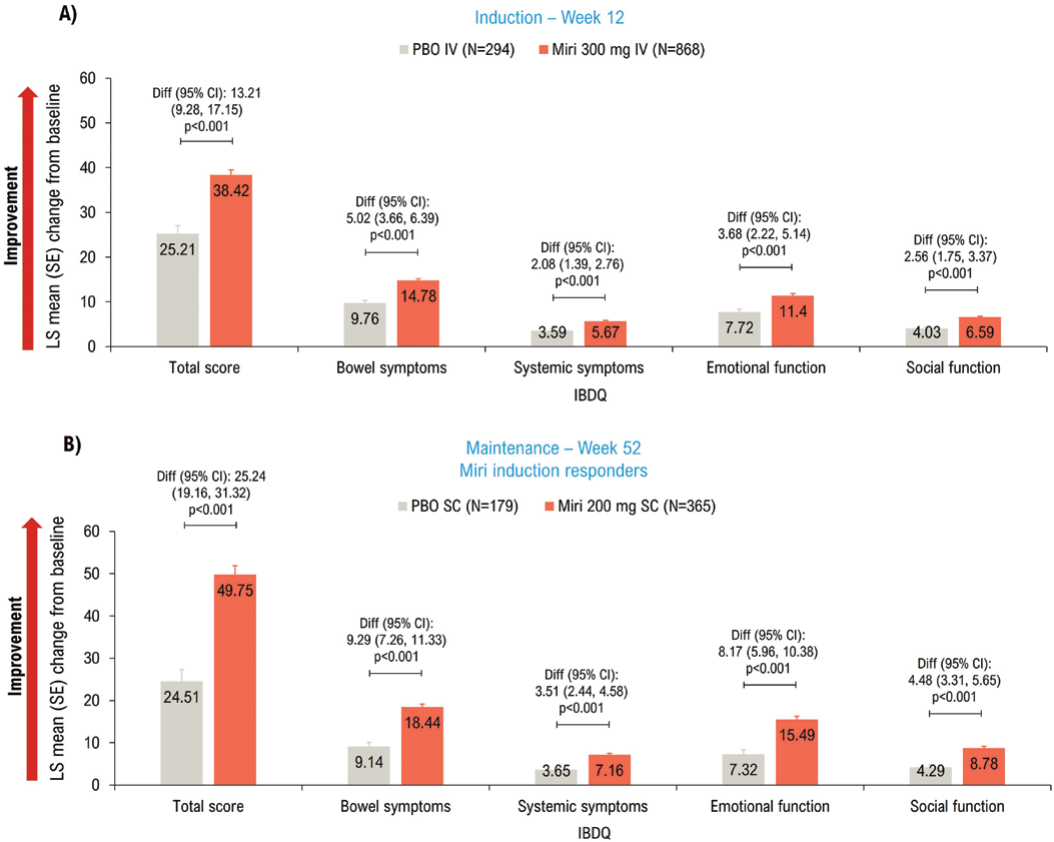
LSM change from baseline (ANCOVA with mBOCF) in IBDQ total and domain scores—mITT. ANCOVA, analysis of covariance; CI, confidence interval; Diff, difference; IBDQ, Inflammatory Bowel Disease Questionnaire; IV, intravenous; LSM, least squares mean; mBOCF, modified baseline observation carried forward; miri, mirikizumab; mITT, modified intent-to-treat; PBO, placebo; SC, subcutaneous; SE, standard error.

IBDQ total score^14^ improved significantly (*P* < .001) in the mirikizumab versus the placebo group at Week 12 (LS mean difference [95% CI]: 13.21 [9.28, 17.15]; Figure 1A) and Week 52 (LS mean difference [95% CI]: 25.24 [19.16, 31.32]; Figure 1B). Mirikizumab-treated patients showed significant improvement across all IBDQ domain scores compared to placebo at Weeks 12 and 52 (*P* < .001; Figure 1). The proportion of patients who achieved IBDQ response was significantly (*P* < .001) higher in the mirikizumab group versus the placebo group at Week 12 (72.7% [*n* = 631] versus 55.8% [*n* = 164]; risk difference [95% CI]: 17.1 [10.7, 23.5]) and Week 52 (79.2% [*n* = 289] versus 49.2% [*n* = 88]; risk difference [95% CI]: 29.5 [21.0, 37.9]). IBDQ remission was achieved in a significantly (*P* < .001) greater proportion of patients treated with mirikizumab than placebo at Week 12 (57.5% [*n* = 499] versus 39.8% [*n* = 117]; risk difference [95% CI]: 18.1 [11.8, 24.4]) and Week 52 (72.3% [*n* = 264] versus 43.0% [*n* = 77]; risk difference [95% CI]: 28.5 [20.1, 37.0]).

#### Medical Outcomes Study 36-Item Short Form Health Survey

**Figure 2.**
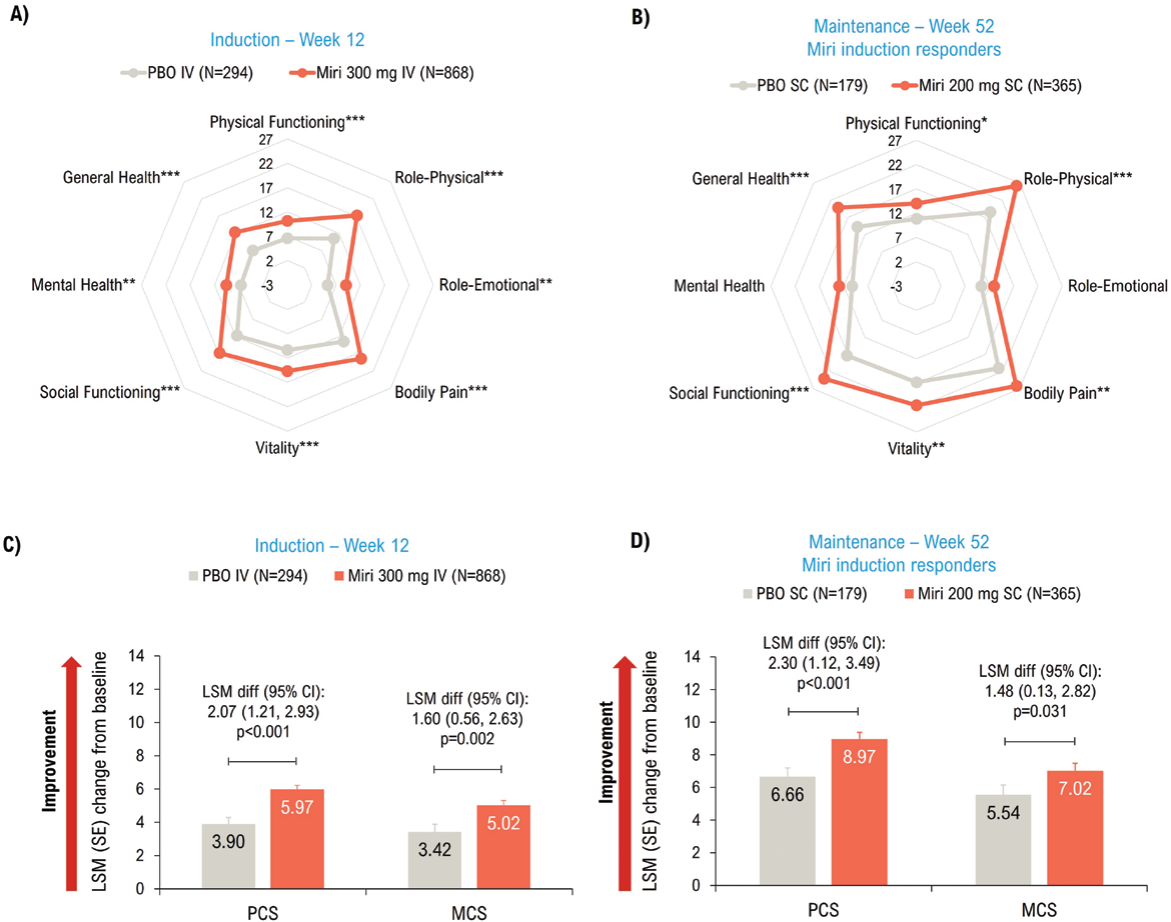
LSM change from baseline (ANCOVA with mBOCF) in SF-36 (A and B) Domain scores; (C and D) Summary scores—mITT. *P < .05, **P < .01, ***P < .001 vs PBO. ANCOVA, analysis of covariance; CI, confidence interval; diff, difference; IV, intravenous; LSM, least squares mean; MCS, Mental Component Summary; mBOCF, modified baseline observation carried forward; miri, mirikizumab; mITT, modified intent-to-treat population; PBO, placebo; PCS, Physical Component Summary; SC, subcutaneous; SE, standard error; SF-36, 36-Item Short Form Health Survey.

At Week 12, significant improvements in PCS (*P* < .001), MCS (*P* = .002), and all domain scores (*P* < .01) of SF-36 were achieved with mirikizumab versus placebo (Figure 2A, C). Improvement in SF-36 scores was sustained through the maintenance period, with significant improvements seen in the mirikizumab group compared to placebo in PCS (*P* < .001), MCS (*P* = .031), and 6/8 domain scores (physical functioning, role-physical, bodily pain, vitality, social functioning, and general health; all *P* < .05) at Week 52 (Figure 2B, D).

PCS MCID response was achieved in a greater proportion of mirikizumab-treated patients versus placebo at Week 12 (50.6% [*n* = 439] versus 41.5% [*n* = 122]; risk difference [95% CI]: 8.9 [2.4, 15.4]; *P* = .008) and Week 52 (61.9% [*n* = 226] versus 36.9% [*n* = 66]; risk difference [95% CI]: 25.0 (16.2, 33.7); *P* < .001). A similar pattern was seen for MCS MCID response rates at Week 12 (44.2% [*n* = 384] versus 37.8% [*n* = 111]; risk difference [95% CI]: 6.8 [0.4, 13.3]; *P* = .040) and Week 52 (51.2% [*n* = 187] versus 34.6% [*n* = 62]; risk difference [95% CI]: 16.2 [7.5, 24.9]; *P* < .001).

#### EQ-5D-5L VAS

**Figure 3.**
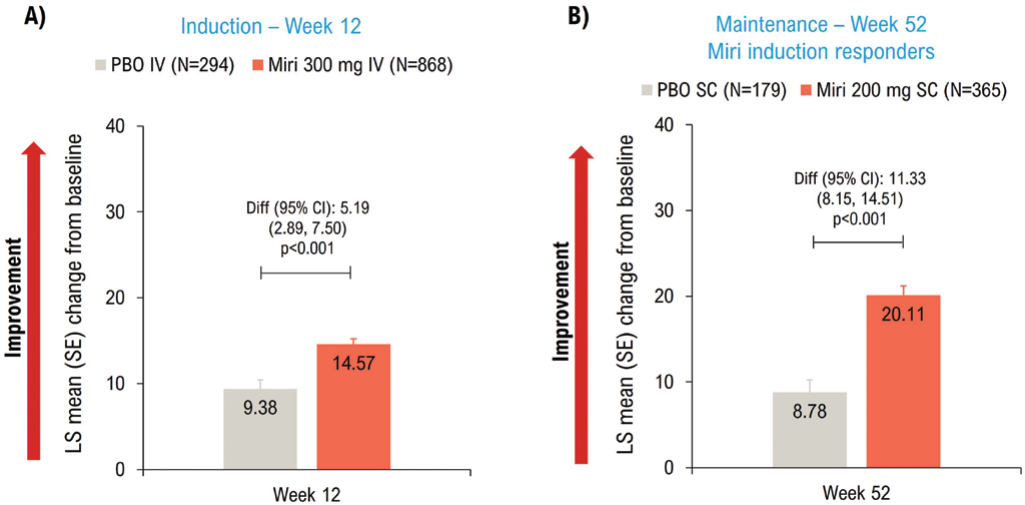
LSM change from baseline (ANCOVA with mBOCF) in EQ-5D-5L VAS—mITT. ANCOVA, analysis of covariance; CI, confidence interval; Diff, difference; IV, intravenous; LSM, least squares mean; mBOCF, modified baseline observation carried forward; miri, mirikizumab; mITT, modified intent-to-treat; PBO, placebo; SC, subcutaneous; SE, standard error; VAS, visual analog scale.

EQ-5D-5L VAS scores improved significantly (*P* < .001) in the mirikizumab group compared to placebo at Weeks 12 (Figure 3A) and 52 (Figure 3B).

#### Work Productivity and Activity Impairment Questionnaire:UC

**Figure 4.**
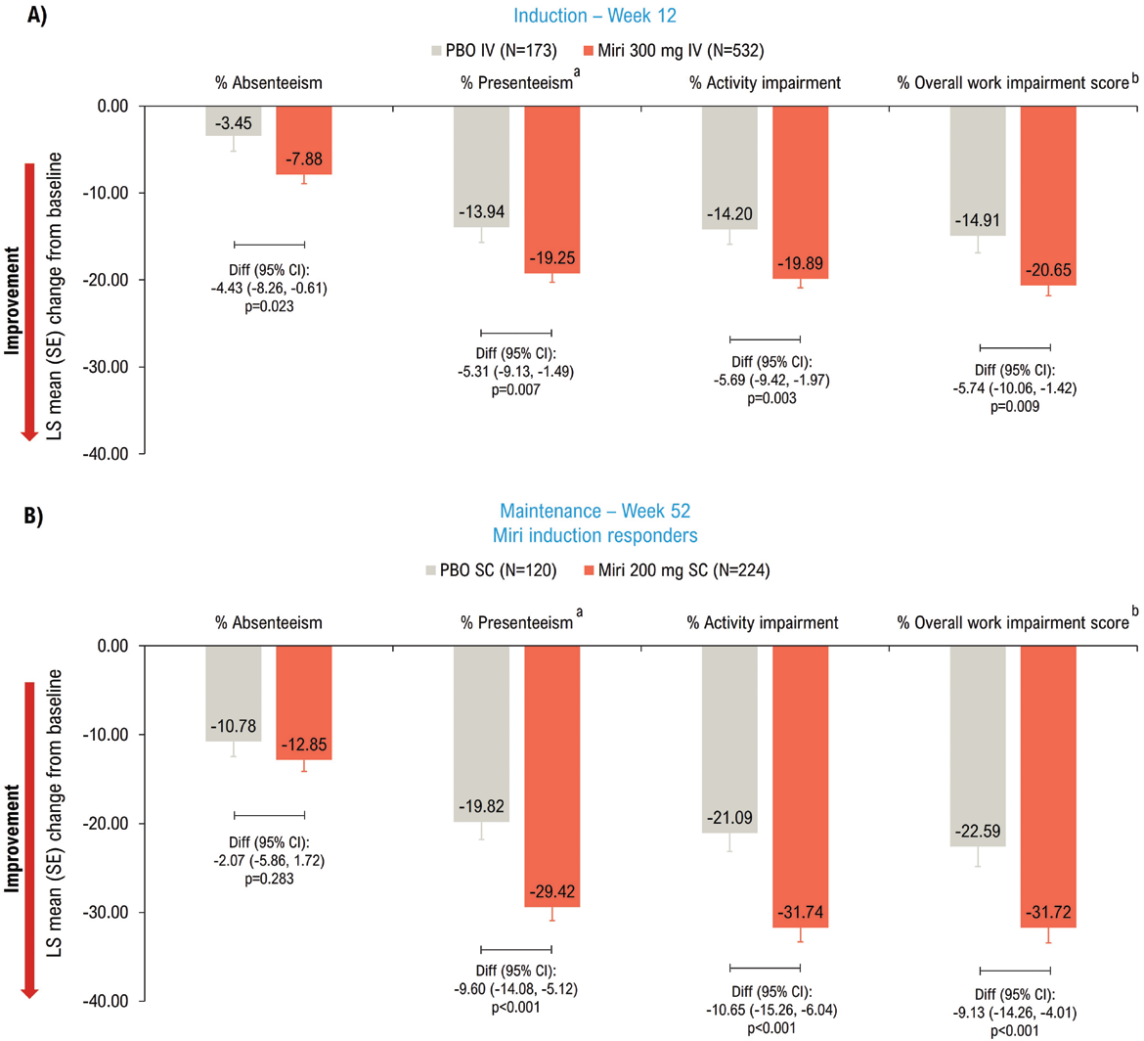
LSM change from baseline (ANCOVA with mBOCF) in WPAI:UC—mITT. ^a^Reduced productivity while at work; ^b^Overall work impairment score is an aggregate of absenteeism and presenteeism. ANCOVA, analysis of covariance; CI, confidence interval; Diff, difference; IV, intravenous; LSM, least squares mean; mBOCF, modified baseline observation carried forward; miri, mirikizumab; mITT, modified intent-to-treat; PBO, placebo; SC, subcutaneous; SE, standard error; WPAI:UC, Work Productivity and Activity Impairment Questionnaire Ulcerative Colitis.

At Week 12, mirikizumab-treated patients showed a significant improvement compared to placebo in all 4 scores: absenteeism (*P* = .023), presenteeism (*P* = .007), activity impairment (*P* = .003), and overall work impairment (*P* = .009) (Figure 4A). This significant improvement was sustained at Week 52 (*P* < .001) for 3 out of 4 scores: presenteeism, activity impairment, and overall work impairment; absenteeism was similar between the two groups at Week 52 (*P* = .283) (Figure 4B).

#### Patient Global Rating of Severity

**Figure 5.**
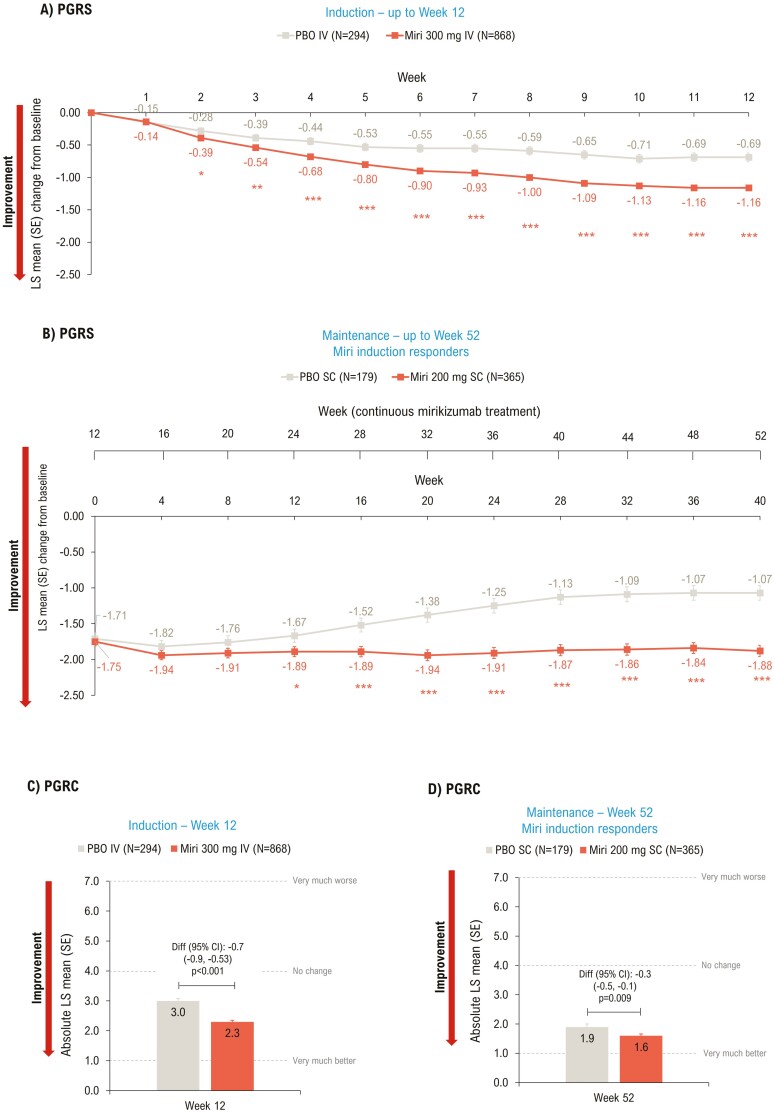
LSM change from baseline (ANCOVA with mBOCF) in PGRS (A and B) and absolute LSM (ANCOVA as observed) PGRC (C and D)—mITT. *P < .05, **P < .01, ***P < .001 vs PBO. ANCOVA, analysis of covariance; CI, confidence interval; diff, difference; IV, intravenous; LSM, least squares mean; mBOCF, modified baseline observation carried forward; miri, mirikizumab; mITT, modified intent-to-treat population; PBO, placebo; PGRS, Patient’s Global Rating of Severity; PGRC, Patient’s Global Rating of Change; SC, subcutaneous; SE, standard error; W, week.

In the induction study, significant improvement in PGRS scores was observed in the mirikizumab group versus placebo as early as Week 2 (*P* < .05) and scores continued to improve up to Week 12 (−1.16 vs −0.69; *P* < .001) (Figure 5A). Improvement in PGRS scores in mirikizumab- versus placebo-treated patients was sustained throughout the maintenance period, with significant improvements seen at Week 52 (−1.88 vs −1.07; *P* < .001) (Figure 5B).

#### Patient Global Rating of Change

Patients in the mirikizumab group showed significant improvement in PGRC at Weeks 12 (*P* < .001; Figure 5C) and 52 (*P* = .009; Figure 5D) compared to placebo.

## Discussion

In the phase 3 LUCENT-1 and LUCENT-2 studies, mirikizumab showed significant and clinically meaningful improvement in QoL of patients with moderately-to-severely active UC. There is a lack of validated PROs specifically for UC; however, our study assessed improvement in both disease-related (IBDQ and WPAI:UC) and diverse (SF-36, EQ-5D-5L, PGRS, and PGRC) PROs. The improvements seen in these outcomes during the induction study (Week 12) were sustained through the maintenance period (Week 40; ie, 52 weeks of continuous therapy).

IBDQ is commonly used to assess disease-specific QoL.^26^ In the current study, ~80% of mirikizumab-treated patients who were induction responders at Week 12 achieved IBDQ response, and ~70% of patients achieved IBDQ remission at Week 52. A recent study evaluating sensitivity of IBDQ reported treatment efficacy as a positive predictor of IBDQ outcomes and showed strong association between IBDQ and changes in clinical health in response to treatment.^27^

SF-36 has been used in several UC clinical trials to detect changes in QoL with respect to treatment efficacy^28–32^; treatments that induce and maintain remission have shown to normalize and restore mental and physical well-being.^33^ Clinical remission rates in LUCENT-1 (Week 12) and LUCENT-2 (Week 40) studies were significantly higher in the mirikizumab group versus placebo.^14^ Consistent with these findings, a significantly greater proportion of mirikizumab-treated patients achieved clinically important improvement in PCS and MCS scores at Weeks 12 and 52 compared to placebo.

UC-related symptoms increase absenteeism and have a negative impact on patients’ productivity at work (i.e., presenteeism), leading to unemployment and use of disability compensation.^34,35^ A recent systematic review reported that UC is associated with high annual absenteeism costs in North America ($1443 per person; range: $85–$2350) and Europe ($2394 per person; range: $651–$5992).^36^ In comparison to patients without IBD, patients with UC reported higher work loss-related costs per patient per year ($5307 vs $3165; *P* < .001) in the United States.^37^ Mirikizumab treatment significantly improved all WPAI:UC scores, except absenteeism, at Week 52 versus placebo. The impact on absenteeism may be explained by the majority of patients reporting zero absence due to UC in the past 7 days, findings in agreement with previously reported studies.^34^

In the current study, EQ-5D-5L VAS^38^ scores improved significantly with mirikizumab versus placebo during the induction and maintenance studies. Compared to placebo, mirikizumab-treated patients reported significant improvement in PGRS scores as early as Week 2, with ~2-point improvement in mean PGRS score at Week 52. Similarly, mirikizumab significantly improved mean PGRC scores at Weeks 12 and 52 versus placebo.

Consistent with our study findings, other phase 3 studies evaluating advanced therapies for UC, have also demonstrated improvement in QoL.^29–32,39^

In addition to clinical remission, treatment effect needs to be assessed from the patients’ perspectives. The STRIDE-II recommendations include absence of disability and normalized health-related QoL as long-term treatment targets for moderate-to-severe UC and suggest changing treatment if these targets are not achieved.^8^ The current study utilized a broad range of PROs to evaluate the effect of mirikizumab on various dimensions of QoL including UC symptoms, change in severity of symptoms, physical and mental well-being, and work productivity and daily activities.

## Conclusions

Mirikizumab demonstrated significant and clinically important improvement across all domains of QoL in patients with moderately-to-severely active UC during LUCENT-1 induction and LUCENT-2 maintenance studies.

## Supplementary Material

otad070_suppl_Supplementary_Tables_1

## Data Availability

Lilly provides access to all individual participant data collected during the trial, after anonymization, with the exception of pharmacokinetic or genetic data. Data are available to request 6 months after the indication studied has been approved in the United States and European Union and after primary publication acceptance, whichever is later. No expiration date of data requests is currently set once data are made available. Access is provided after a proposal has been approved by an independent review committee identified for this purpose and after receipt of a signed data sharing agreement. Data and documents, including the study protocol, statistical analysis plan, clinical study report, blank, or annotated case report forms, will be provided in a secure data sharing environment. For details on submitting a request, see the instructions provided at www.vivli.org.
