# Supplementary material for: Mirikizumab Improves Quality of Life in Patients With Moderately-to-Severely Active Ulcerative Colitis: Results From the Phase 3 LUCENT-1 Induction and LUCENT-2 Maintenance Studies
Source: Crohns Colitis 360. 2023 Nov 7;5(4):otad070. doi: 10.1093/crocol/otad070 (PMC10684049; doi:10.1093/crocol/otad070)
Supplement: otad070_suppl_Supplementary_Tables_1 [file otad070_suppl_supplementary_tables_1.docx]

**Mirikizumab Improves Quality of Life in Patients With Moderately-to-Severely Active Ulcerative Colitis: Results From the Phase 3 LUCENT-1 Induction and LUCENT-2 Maintenance Studies**

**Supplementary Table 1. Detailed description of patient-reported outcome measures**

| **Patient-reported QoL measures (Range)** | |
| --- | --- |
| IBDQ^1,2^ | The IBDQ questionnaire contains 32 items distributed across 4 domains/subscores: i) bowel symptoms, ii) systemic symptoms, iii) emotional function, and iv) social function. The items are graded on a 7-point Likert scale (7=“not a problem at all” and 1=“a very severe problem”) over a recall period of last 2 weeks. Total score (32–224) is the sum of the 4 domains/subscores. Higher score indicates better QoL. |
| SF-36 Version 2^3,4^ | The 36-item questionnaire measures 2 overall summary scores (range: 0–100; PCS and MCS) and 8 health domain scores (range: 0–100; physical functioning, role-physical, role-emotional, bodily pain, vitality, social functioning, mental health, and general health) over a recall period of last 4 weeks.  Higher scores indicate better function and/or health. |
| EQ-5D-5L VAS^5,6^ | The questionnaire assesses patients’ current health status using visual analogue scale (VAS; range: 0=“worst imaginable health state” to 100=“best imaginable health state”).  Higher score indicates better health state. |
| WPAI:UC^7,8^ | WPAI:UC measures the impact of UC on work productivity and regular activities during the past 7 days. Four scores (absenteeism, presenteeism, activity impairment, and overall work impairment) are calculated as impairment percentages based on response to following 6 items:  i] employment status; ii] hours missed from work due to the disease; iii] hours missed from work due to other reasons; iv] hours actually worked; v] productivity affected by the disease while working; and vi] productivity affected by the disease while performing regular unpaid activities.  Patients who are employed will complete the absenteeism, presenteeism, and work productivity loss questions, and all patients will complete the activity impairment question.  Higher scores indicate greater impairment and less productivity. |
| **Patient-reported outcomes (Range)** | |
| PGRS (1–6) | The 1-item questionnaire assesses the patients’ rating of their disease symptom severity over the past 24 hours using a 6-point scale: 1=“no symptoms” and 6=“very severe”. |
| PGRC (1–7) | Assesses the patients’ rating of change in their symptom(s). Responses are graded on a 7-point Likert scale (1=“very much better,” 4=“no change,” and 7=“very much worse”). |

IBDQ, Inflammatory Bowel Disease Questionnaire; MCS, Mental Component Summary; PCS, Physical Component Summary; PGRC, Patient Global Rating of Change; PGRS, Patient Global Rating of Severity; QoL, quality of life; SF-36, Medical Outcomes Study 36-Item Short Form Health Survey; UC, ulcerative colitis; VAS, visual analogue scale; WPAI, Work Productivity and Activity Impairment Questionnaire.

1. Irvine EJ, Zhou Q, Thompson AK. The short inflammatory bowel disease questionnaire: A quality of life instrument for community physicians managing inflammatory bowel disease. Ccrpt investigators. Canadian crohn's relapse prevention trial. Am J Gastroenterol 1996;91:1571–8.

2. Guyatt G, Mitchell A, Irvine EJ*, et al.* A new measure of health status for clinical trials in inflammatory bowel disease. Gastroenterology 1989;96:804–10.

3. Ware JE, Jr., Sherbourne CD. The MOS 36-item short-form health survey (SF-36). I. Conceptual framework and item selection. Med Care 1992;30:473–83.

4. Maruish M, Kosinski M, et al. User’s manual for the SF-36v2 health survey. In, 2011.

5. Herdman M, Gudex C, Lloyd A*, et al.* Development and preliminary testing of the new five-level version of EQ-5D (EQ-5D-5L). Qual Life Res 2011;20:1727–36.

6. Euroqol research foundation. EQ-5D-5L user guide, 2019. Available from: <Https://euroqol.Org/publications/user-guides>. Accessed 12 July 2022.

7. Reilly MC, Zbrozek AS, Dukes EM. The validity and reproducibility of a work productivity and activity impairment instrument. Pharmacoeconomics 1993;4:353–65.

8. [Reilly associates] Reilly associates WPAI scoring. Available at: <Http://www.Reillyassociates.Net/wpai_scoring.Html>. Accessed 12 July 2022.
